# Role of Breast-Conserving Surgery on the National Health System Economy From and to SARS-COVID-19 Era

**DOI:** 10.3389/fsurg.2021.705174

**Published:** 2022-01-25

**Authors:** Oreste Claudio Buonomo, Danilo Vinci, Gerardo De Carolis, Marco Pellicciaro, Francesco Petracca, Amir Sadri, Chiara Buonomo, Mario Dauri, Gianluca Vanni

**Affiliations:** ^1^Breast Unit, Department of Surgical Science, PTV Policlinico Tor Vergata University, Rome, Italy; ^2^Health Management, Fondazione PTV, Rome, Italy; ^3^CeRGAS (Centre for Research on Health and Social Care Management), SDA Bocconi School of Management, Milan, Italy; ^4^Plastic Surgery, Great Ormond Hospital for Children NHS Foundation Trust, London, United Kingdom; ^5^Department of Emergency and Admission, Critical Care Medicine, Pain Medicine and Anesthetic Science, Policlinico Tor Vergata University, Rome, Italy

**Keywords:** SARS-CoV-2, COVID-19, healthcare cost, day-surgery, cost saving, breast conserving surgery, awake surgery

## Abstract

Day surgery breast-conserving surgery (DS-BCS) is a surgical approach applied in many specialized breast surgery departments. This study demonstrates the benefits of this approach from the perspectives of patients and of the Hospital/National Health System compared to ordinary breast-conserving surgery (ORD-BCS) under general anesthetic. A comparison of costs and diagnosis-related group (DRG) reimbursement demonstrated improved cost-effectiveness in DS-BCS compared to ORD-BCS.

## Introduction

Day surgery is becoming increasingly utilized in many healthcare systems. It reduces patient waiting time by increasing patient throughput while ensuring high quality care. Day surgery can also significantly reduce the cost of healthcare (1, 2). Traditionally, a minimum of 2 days of inpatient stay is required for breast surgical procedures, such as quadrantectomy, and this has been attributed to general anesthesia.

It has been previously demonstrated that the status of axillary lymph nodes is safely assessed by sentinel node biopsy (SLNB), particularly when approaching early-stage breast cancer (3, 4). Reduced postoperative pain, absence of drainage from the axilla, decreased percentage of neurovascular complication and lymphedema are some of the many advantages of SLNB.

Opioid-based general anesthesia and perioperative analgesia represent the main trigger for postoperative nausea, vomiting, respiratory problems, urinary retention, ileus, and hyperalgesia. Moreover, some studies have shown a higher probability of metastasis related to the choice of anesthetic setting. (5, 6). Previous reports have shown no intraoperative pain during an awake patient quadrantectomy procedure with ropivacaine infiltration (7, 8).

Many protocols have demonstrated that regional anesthesia techniques such as thoracic paravertebral block (TPVB), pectoral nerve block (PECS), erector spinae plane (ESP) block, and serratus anterior plane (SAP) result in a reduction in opioid usage during the postoperative period (9, 10).

We have introduced the concept of awake breast surgery during the COVID-19 pandemic as a means of reducing the incidence of viral infection and maximizing the utility of hospital service during a period of intense pressure (11–13).

Based on our report (11), financial analysis of awake breast surgery would be beneficial to help improve the current model of care for patients with breast cancer that require wide local excision.

Based on this, in a health context in which 70% of procedures have been carried out with the awake approach and 30% of them with a conventional approach, it is interesting to make a forecast of income in a 1-year time lapse and compare it to the 30% awake BCS rate before the COVID-19 pandemic.

The objective of this study is to perform a cost analysis of breast surgery undertaken when 70% of cases were performed using regional anesthesia vs. 30% with conventional general anesthesia. This would be compared to the standard of care before the pandemic when the division of regional vs. general anesthesia was reversed.

## Materials and Methods

### Study Design

In this study, we retrospectively enrolled all patients undergoing breast-conserving surgery from January to March 2020. From this cohort, patients undergoing breast conserving surgery and sentinel lymph node biopsy were grouped according to day surgery (DS-BCS) or ordinary surgery (ORD-BCS).

Patients have been assessed pre-operatively by mammography, ultrasound, or MRCP. Lymphoscintigraphy was performed externally the day before surgery. All malignant cases that were suitable for breast-conserving surgery were included. Exclusion criteria were: men, pregnancy, pure breast reconstruction (BR) surgical procedures, and benign disease.

In order to discharge day surgery patients, all discharge criteria (Table 1) had to be met; otherwise, they were admitted to an inpatient ward for a one-night stay.

**Table 1:** Discharge criteria.

**Discharge criteria**
Stable vital signs
Alert and orientated
Absence of respiratory distress
Pain controlled
No bleeding (drainage < 100 cc in 24/h)
Steady gait, no dizziness or meets preoperative level

Hospital data concerning the number of patients in the year 2019 were collected in order to do a forecast of income with the adoption of such a greater day surgery surgical approach.

### Inclusion and Exclusion Criteria

Patients between 18 and 90 years old whose physical status corresponded to the American Society of Anesthesiologists (ASA) physical status classification system (14) grades I-II were enrolled for awake breast-conserving surgery in a day surgical setting. ASA grade III patients who presented with stable clinical status and well-controlled comorbidities could be enrolled for awake BCS. Older patients who, during the considered period, gave consent to awake breast surgery in the day surgical setting were included.

Patients who did not give their informed consent to the awake procedure, ASA grade IV patients, or patients with preoperative indication to radical mastectomy were excluded.

### Hospital Cost and NHS Cost

A cost analysis for both DS-BCS and ORD-BCS was carried out considering both fixed costs, such as surgical instruments, and variable costs (operating theater and ward bed). Bed cost has been modified according to the length of stay in the case of an overnight stay.

Operating time cost was calculated as the actual cost per hour. For DS-BCS, this was calculated as € 240/h and € 600/h for ORD-BCS (the difference in cost is due to the number of nurses, operating staff, devices, and operating room length of usage). All costs are updated for the 2020/2021 health costs (15).

Ward bed cost was € 150 for DS-BCS and € 605/day for ORD-BCS; for patients who required longer stay, the daily cost was multiplied by the number of inpatient days (15).

Total cost is given by the sum of the operating theater time and ward bed multiplied by the number of procedures performed within each setting. Hospital coding and accounts services provided all data on cost.

In order to analyze the financial cost better from a National Health System perspective, a comparison between DS-BCS diagnosis-related group (DRG) and ORD-BCS (DRG) was performed. The refunded cost was € 2831.47 for ORD-BCS and € 1,362 for DS-BCS, and these represent the maximum tariffs paid to hospitals with a flat fee (www.gazzettaufficiale.it) according to the current Legislative Decree of October 18, 2012.

### Endpoints

The primary outcome was to evaluate the total cost difference between DS-BCS and ORD-BCS assuming a zero 30-day readmission rate. This assumption represents one limitation of this study. Readmission is defined as hospitalization occurring within 30 days from discharge and lasting at least 24 h.

The secondary outcome was to make a forecast of net income; adopting the awake surgery approach as the main operating setting. This was the standard practice in March 2020 when 70% of cases were carried out as awake surgery in order to reduce hospitalization and increase the number of oncologic patients who could have access to needed surgical procedures.

### Statistical Analysis

We calculated means and ranges for continuous variables. Differences between the two groups were assessed by *t*-test. Categorical data were recoded into numbers and percentages. Fisher's exact test was performed to analyze dichotomous variables such as different surgical procedures. A *p*-value < 0.05 was necessary for a variable to be considered statistically significant. SPSS statistical package version 23.0 (SPSS Inc., Chicago, IL, United States) was used to perform the statistical analysis.

## Results

A total of 56 cases were identified during the study period. Thirty-nine (70%) were eligible for day surgery cases [female 100%, mean age = 71.56 years (SD = 7.8), mean weight 62.15 (SD = 9.73) kg]. The remaining patients (17) who did not satisfy the day surgery criteria were included in the ordinary surgical procedure group [female 100%; mean age = 67.82 (SD = 8.4), mean weight = 66.76 kg (SD = 6.8), *p* = 0.1]. Characteristics of both day surgery and ordinary BCS patients, ASA scores, and comorbidities are listed in Table 2. No major complications were reported. No statistically significant difference in patient characteristics between the two groups was found (*p* > 0.05).

**Table 2:** Patient characteristics of day surgery breast-conserving surgery (DS-BCS) vs. ordinary breast-conserving surgery (ORD-BCS).

**Variables**	**Day surgery BCS**	**Ordinary BCS**	***p*-value**
Number of patients	39	17	-
Age (years)	71, 56 (SD = 7, 8)	67, 82 (SD = 8, 4)	0, 1
Weight (Kg)	62, 15 (SD = 9, 73)	66, 76 (SD = 6, 8)	0, 08
ASA Score			0, 5
ASA grade 1	18 (46%)	6 (36%)	-
ASA grade 2	10 (26%)	7 (41%)	-
ASA grade 3	11 (28%)	4 (23%)	-
Major comorbidities			
Cardiovascular disease	3 (7, 6%)	1 (5, 8%)	0, 78
CKD	2 (5, 1%)	0	0,77
Respiratory disorders	1 (2, 5%)	0	0, 56
Diabetes	2 (5, 1%)	1 (5, 8%)	1

Day surgery BCS resulted in a total hospital expense of € 30,703.53 [mean tariff paid per patient: € 784.42 (SD = 12.58)], while ordinary BCS resulted in a total hospital expense of € 5,3950.89 [mean tariff paid per patient: € 3,158.76 (SD = 53.76)] (*p* = 0.001) (Table 3).

**Table 3:** Composite cost evaluation.

**Variables**	**Day surgery BCS**	**Ordinary BCS**	***p*-value**
Number of patients	39	17	-
Hospital cost per patient	784, 42 (SD = 12, 58)	3158,76 (SD = 53, 76)	0, 001
Operative time cost	449, 53 (SD = 55, 14)	1527, 35 (SD = 198, 76)	0, 001
Ward cost	150 (SD = 20, 07)	605 (SD = 48, 28)	0, 001
DRG reimbursement per patient	1362	2831, 47	0, 001
Total hospital cost	30703, 53	53950, 89	0, 001
Total DRG Reimbursement	53118	48135	0, 001

Overall NHS costs for day surgery and ordinary surgery were € 53,118 and 48,135, respectively (*p* = 0.001). Mean DS-BCS DRG was € 1,362 vs. € 2,831.47 for BCS-ORD DRG, resulting in a difference (Δ DRG = € 2,831.47– € 1,362) of € 1,469.47 per inpatient procedure and, thus, representing a total overcharge for the Italian NHS of € 24,981 (€ 1,469.47 × 17 ordinary BCS) when day surgical regimen was not considered; this is limited to 2 months (Table 3).

The annual forecast of net income adopting the day surgical awake surgery (70% DS-BCS vs. 30% ORD-BCS) transformed a total annual loss of € 21,431.67 into a net income of € 99,591.43 (Table 4).

**Table 4:** One-year income forecast.

**Operative setting percentage**	**Net income**
30% DS 70% ORD	−21.431, 67
70% DS 30% ORD	+99.591, 43

## Discussion

The search for less invasive treatment of breast cancer is still ongoing. Sentinel node biopsy and wide local excision under local anesthesia are important steps toward optimal results. We are witnessing a more conservative surgical approach to breast cancer. Breast-conserving surgery is supersedingmastectomy.

Immunosuppression represents the physiological response to stress. This may represent a risk for a patient in the perioperative setting. Therefore, one of the most important objectives for patient care is the reduction of perioperative stress (16, 17). Lifestyle factors account for a small (at most 30%) percentage of cytotoxic activity that might become an additional biomarker to consider in a lifestyle intervention for cancer prevention. In a trial for women who had breast cancer more than 5 years before the intervention, it has been demonstrated that cytotoxic activity can be modified by changing several lifestyle factors (18, 19).

The probability of metastases and tumor progression can be increased by blood lymphocyte cytotoxic activity reduction (20, 21). Moreover, surgical site infection (SSI) is predicated by decreased immune function. Minimally invasive techniques have a reduced impact on immune function. However, the protective role of minimally invasive techniques in early lymphocyte response has not been properly demonstrated (7). As reported by Pompeo et al. (22) and Roselli et al. (23), the choice of a general anesthesia setting could influence the immune system; hence, awake breast surgery, avoiding general anesthesia, can positively interfere with postoperative lymphocyte response. Patients' quality of life is positively affected by day surgical BCS, because of shorter hospitalization and early return to normal activities. Furthermore, the day surgical setting is fundamental for better utilization of limited healthcare resources.

To our knowledge, this is the first cost analysis to evaluate the benefits of BCS in day surgery both on the hospital and on the NHS in Italy reported in the literature. Hospital costs resulted in € 784.42 per patient in a day surgical setting and € 3,158.76 for an ordinary setting. Prior to the SARS-CoV2 pandemic, the ratio of surgical approaches was 30% for the awake setting and 70% for the general anesthesia setting. In March 2020, the ratio completely reversed with a 70% awake setting, transforming a loss of € 3,572 into a profit of € 16,599 during 2 months. Furthermore, the ordinary BCS constitutes an important financial burden for NHS. In the Lazio region (Italy), the mean reimbursement for day surgery and ordinary BCS reaches € 1,362 and € 2,831.5, respectively, resulting in NHS mean overcharge of € 1,469.47 for each inpatient BCS. Interestingly, making a 1-year forecast of such a change in the operative setting DS-BCS could represent a prospective profit of € 99,591.4 instead of a loss of € 2,1431.7 when considering BCS inpatient (total DRG reimbursement–hospital cost).

Considering the social impact of breast cancer and the growing importance breast-conserving surgery has acquired during the years, it is rational to analyze and consider the financial burden for healthcare services. We performed a cost effectiveness evaluation by a complete cost analysis that took into account surgery-related costs as well as DRG refunded fees, providing a complete financial assessment that takes into account both local tertiary care centers and NHS.

Thus, we can conclude that considering clinical benefits for patients and financial benefits for hospitals, the awake day surgical approach must always be considered and promoted in well-selected centers. Cost savings that can be achieved could be used for investments in research and patient care. This strategy adopted at our breast unit should be performed routinely and not only during the emergency period. Further larger controlled scale trials are needed to establish safety and more robust cost predictions.

## Data Availability Statement

The original contributions presented in the study are included in the article/supplementary materials, further inquiries can be directed to the corresponding author/s.

## Ethics Statement

The manuscript was approved by the local Ethical Committee of the Fondazione Policlinico Tor Vergata (reference 122/20). Due to retrospective analysis and anonymous data analysis, written informed consent to participate in this study was not provided in accordance with the national legislation and institutional requirements.

## Author Contributions

OB, DV, MP, and GV analyzed the data and wrote the manuscript. GD acquired the economic data. MP performed the statistical analysis. FP analyzed and performed the economic cost evaluation and economic forecast. AS, CB, and MD reviewed the manuscript. All the co-authors gave a contribution to the article and approval to the submitted version.

## Conflict of Interest

The authors declare that the research was conducted in the absence of any commercial or financial relationships that could be construed as a potential conflict of interest.

## Publisher's Note

All claims expressed in this article are solely those of the authors and do not necessarily represent those of their affiliated organizations, or those of the publisher, the editors and the reviewers. Any product that may be evaluated in this article, or claim that may be made by its manufacturer, is not guaranteed or endorsed by the publisher.
